# Clinical value of Vitamin-D combined with budesonide/formoterol and theophylline sodium glycinate sustained-release tablets in the treatment of chronic obstructive pulmonary disease patients

**DOI:** 10.12669/pjms.40.7.9495

**Published:** 2024-08

**Authors:** Xiuyuan Ni, Shigeng Zhou, Congling Wang, Shengheng Chen, Jingdan Hu, Shengjing Zhang

**Affiliations:** 1 Xiuyuan Ni Department of Respiratory, Wenzhou Geriatric Hospital, Wenzhou city, Zhejiang Province 325000, P.R. China; 2 Shigeng Zhou Department of Respiratory, Wenzhou Geriatric Hospital, Wenzhou city, Zhejiang Province 325000, P.R. China; 3 Congling Wang Department of Respiratory, Wenzhou Geriatric Hospital, Wenzhou city, Zhejiang Province 325000, P.R. China; 4 Shengheng Chen Department of Respiratory, Wenzhou Geriatric Hospital, Wenzhou city, Zhejiang Province 325000, P.R. China; 5 Jingdan Hu Department of Respiratory, Wenzhou Geriatric Hospital, Wenzhou city, Zhejiang Province 325000, P.R. China; 6 Shengjing Zhang Department of Geriatric, Zhejiang Chinese Medical University, Affiliated Wenzhou Hospital of Traditional Chinese Medicine, 9 Jiaowei Street, Wenzhou City, Zhejiang Province 325000, P.R. China

**Keywords:** Budesonide/formoterol, Chronic obstructive pulmonary disease, Theophylline sodium glycinate sustained-release tablets, Vitamin-D

## Abstract

**Objective:**

To explore the clinical value of Vitamin-D combined with budesonide/formoterol (BF) and theophylline sodium glycinate (TSG) sustained-release tablets in the treatment of patients with chronic obstructive pulmonary disease (COPD).

**Methods:**

Medical records of 114 patients with CODP, treated in Wenzhou Geriatric Hospital from October 2020 to February 2023, were retrospectively analyzed. Of them, 59 received treatment with Vitamin-D combined with BF and TSG sustained-release tablets (Group-A), and 55 patients received treatment with BF combined with TSG sustained-release tablets (Group-B). Lung function indicators, blood gas status, inflammatory factors, fractional exhaled nitric oxide (FeNO), and 25-hydroxyvitamin D [25(OH)D] levels before and after the treatment in both groups were collected.

**Results:**

After the treatment, lung function indicators, blood gas status, inflammatory factors, FeNO, and 25 (OH) D levels in both groups were significantly improved compared to pretreatment levels, and were significantly better in the Group-A compared to Group-B (P<0.05).

**Conclusions:**

The combination of Vitamin-D, BF, and TSG sustained-release tablets can effectively regulate the blood gas status of patients with COPD, improve lung function, regulate FeNO and 25 (OH) D, and effectively downregulate the levels of inflammatory factors, thus reducing the degree of inflammatory response.

## INTRODUCTION

Chronic obstructive pulmonary disease (COPD) is a common chronic respiratory system disease^1^ that is mainly characterized by progressive and persistent airflow restriction. It is more common in the middle-aged and elderly population, with the estimated prevalence of GOLD-defined COPD being 37.7% in people aged 65–80 years and 22.2% in people aged 40–64 years.^2^ Patients with COPD often accompanied by varying degrees of difficulty breathing, shortness of breath, phlegm, chronic cough, etc.^1^,^3^ In the absence of timely and effective intervention, COPD may lead to respiratory failure, pulmonary heart disease, etc.^1^-^4^ Therefore, early implementation of safe and effective treatment for patients with COPD is of great significance.^3^,^4^

Common treatment for COPD often includes a combined administration of budesonide, a potent corticosteroid, and formoterol, a long-acting beta2-adrenergic agonist.^5^ This regimen is effective for bronchodilation, and has anti-allergenic, and anti-inflammatory effect.^6^,^7^ Theophylline (1,3-Dimethylxanthine), is another common medication, administered to patients with COPD.^8^,^9^ It inhibits phosphodiesterase and blocks adenosine receptors, alleviates bronchospasm, relaxes bronchial smooth muscle, improves symptoms caused by restricted airway airflow, and is associated with satisfactory compliance.^8^

Theophylline sodium glycinate (TSG) sustained-release tablets are a modified compound of theophylline, which has a more prominent pharmacological effect compared to conventional theophylline.^9^ Current studies have showed that COPD is often accompanied by varying degrees of Vitamin-D deficiency that may cause respiratory muscle weakness, decrease respiratory motility, hinder sputum discharge, and affect lung function.^10^,^11^

In recent years, our hospital has provided treatment for patients with COPD with Vitamin-D combined with budesonide/formoterol (BF) and TSG sustained-release tablets. This retrospective analysis of the treatment status of patients with COPD aimed to provide treatment references for relevant clinical workers.

## METHODS

Clinical records of 114 patients with CODP admitted to Wenzhou Geriatric Hospital from October 2020 to February 2023 were retrospectively analyzed. Fifty-nine patients received treatment with Vitamin-D combined with BF powder inhalers and TSG sustained-release tablets, and were assigned to Group-A, while 55 patients that were treated with BF combined with TSG sustained-release tablets, were assigned to Group-B.

### Ethical Approval:

The ethics committee of Wenzhou Geriatric Hospital approved this study on 2023-Aug-30, No. WLNY-A-2023002.

### Inclusion criteria:

- Patients diagnosed as COPD.^12^
- No additional glucocorticoids or immunosuppressants were taken during the treatment period.
- The clinical data is complete.

### Exclusion criteria:

- Patients with acute exacerbation within the first month prior to inclusion in the study.
- Patients with severe pneumonia, tuberculosis, lung cancer, and asthma.
- Patients with organic lesions such as kidney, heart, and liver.
- Patients with allergic to BF or TSG sustained-release tablet or Vitamin-D (calcitriol).
- Patients with autoimmune diseases and diabetes.

### Treatment:

Both groups received conventional treatments such as antiasthmatic, antispasmodic, and cough relief treatment.

Group-B (patients received BF combined with TSG sustained-release tablets): Patients received BF inhalation powder (AstraZeneca AB, Sweden; Specification: 160 μg/4.5 μg, 60 suction/bottle) 1~2 puffs per actuation, twice daily. In addition, the patients received TSG sustained-release tablets (Jiangsu Pingguang Pharmaceutical Co., Ltd.,Jiangsu, China; Specification: 12 pieces/plate), one tablet each time, twice daily.

Group-A (patients received Vitamin-D combined with BF and TSG sustained-release tablets): Patients received Vitamin-D based on BF and TSG sustained-release tablets. The administration of BF and TSG sustained-release tablets was same as Group-A. Patients orally taken Vitamin-D (calcitriol) (Qingdao Zhengda Pharmaceutical Co., Ltd; Specification: 0.25 μg/tablet) once a day, 0.25 ug/time.

Both groups were treated continuously for eight weeks.

### Outcome measures:

Lung function indicators, including peak expiratory flow (PEF), forced expiratory volume in first second (FEV1), and FEV1/forced vital capacity (FVC) levels, were tested by the MasterScreenpneum O-type lung function detector (Yeager, Germany).

Blood gas status, including oxygen saturation in the arterial blood (SaO_2_), CO_2_ partial pressure (PaCO_2_), and partial pressure of oxygen (PaO_2_) was tested by Rapidlab1265 blood gas analyzer (Siemens, Germany).

Inflammatory factors, such as interleukin-6 (IL-6), tumor necrosis factor-α (TNF -α), and highly sensitive C-reactive protein (hs-CRP). Briefly, 4 ml of fasting venous blood was centrifuged (3500 r/min, 10 minutes) to collect supernatant. TNF-α and IL-6 were measured using enzyme-linked immunosorbent assay; Hs-CRP was measured by immune scattering turbidimetry. The reagent kits were purchased from Wuhan Doctoral Biotechnology Co., Ltd. (Wuhan, China).

Twenty five (OH) D levels were determined by enzyme-linked immunosorbent assay, and the kit was purchased from Wuhan Doctoral Biotechnology Co., Ltd. (Wuhan, China); FeNO was measured using the NIOXFeNO measurement system (Aerochrome, Sweden).

### Statistical analysis:

All data analyses were conducted using SPSS26.0 software (IBM Corp, Armonk, NY, USA). The normality of data was evaluated using the Shapiro Wilk test. The data of normal distribution was represented by mean ± standard deviation, independent sample *t*-test was used for inter group comparison, and paired t-test was used for intra group comparison before and after the treatment. Non-normal distribution data were represented by median and interquartile intervals, and inter group comparisons were conducted using Mann Whitney *U* test. The counting data were represented by the number of cases using Chi-square test. When *P*<0.05, the difference was considered statistically significant.

## RESULTS

A total of 114 patients (58 males and 56 females) were included in this study. Age of the patients ranged from 54 to 83 years, with a mean of 69.95 ± 6.99 years. The course of the disease was 2-12 years, with a median course of 6 (5-8) years. Body mass index (BMI) ranged from 17.9 to 29.5kg/m², with the mean BMI of 23.74 ± 2.86 kg/m². There was no significant difference in baseline data between the two groups (*P*>0.05) (Table-I).

**Table-I:** Comparison of baseline data between two groups.

Group	n	Male/Female	Age (years)	Course of Disease (years)	BMI (kg/m²)
Group-A	59	32/27	69.03±6.73	6 (5-7)	23.97±2.93
Group-B	55	26/29	70.93±7.19	6 (7-9)	23.50±2.80
*χ^2^/t*/Z		0.552	-1.453	-1.360	0.868
P		0.457	0.149	0.174	0.387

Before the treatment, there was no statistically significant difference in lung function indicators between the two groups (*P*>0.05). After the treatment, PEF, FEV_1_, and FEV_1_/FVC in both groups increased compared to before treatment, and were significantly higher in the Group- A compared to Group-B (*P*<0.05) (Table-II).

**Table-II:** Comparison of lung function indicators between two groups.

Time	Group	n	PEF (L/s)	FEV_1_ (L)	FEV_1_/FVC (%)
Before treatment	Group-A	59	1.57±0.40	1.57±0.28	43.32±5.69
	Group-B	55	1.63±0.43	1.61±0.30	42.80±6.71
	t		-0.746	-0.807	0.449
	P		0.457	0.421	0.654
After treatment	Group-A	59	2.26±0.43^a^	2.41±0.33^a^	68.46±6.02^a^
	Group-B	55	1.92±0.46^a^	1.91±0.35^a^	60.31±7.14^a^
	t		4.099	7.731	6.601
	P		<0.001	<0.001	<0.001

***Note:*** Compared with the same Group-Before treatment,

a P<0.05.

Before the treatment, there was no statistically significant difference in blood gas status indicators between the two groups (*P*>0.05). After the treatment, SaO_2_ and PaO_2_ in both groups increased compared to before treatment, and were significantly higher in Group-A patients (*P*<0.05). Post-treatment PaCO_2_ levels decreased compared to pretreatment levels, and were significantly lower in the Group-A compared to Group-B (*P*<0.05) (Table-III).

**Table-III:** Comparison of blood gas status indicators between two groups.

Time	Group	n	SaO_2_ (%)	PaO_2_ (mmHg)	PaCO_2_ (mmHg)
Before treatment	Group-A	59	74.71±8.64	64.44±4.52	55.12±5.61
	Group-B	55	76.27±7.47	63.09±5.22	53.98±6.00
	t		-1.029	1.478	1.045
	P		0.306	0.142	0.298
After treatment	Group-A	59	88.07±6.45^a^	92.31±4.37^a^	38.73±5.03^a^
	Group-B	55	83.44±9.42^a^	89.40±6.04^a^	41.38±5.96^a^
	t		3.079	2.956	-2.575
	P		0.003	0.004	0.011

***Note:*** Compared with the same Group-Before treatment,

a P<0.05.

Pretreatment levels of serum inflammatory factor indicators were comparable in both groups (*P*>0.05). After the treatment, the serum levels of IL-6, TNF-α, and hs-CRP in both groups decreased, and Group- A patients exhibited significantly lower levels of serum inflammatory factor indicators compared to Group-B (*P*<0.05) (Table-IV).

**Table-IV:** Comparison of two groups of inflammatory factors.

Time	Group	n	IL-6 (ng/ml)	TNF-α (ng/ml)	hs-CRP (mg/L)
Before treatment	Group-A	59	35.90±5.46	46.56±8.21	17.24±4.46
	Group-B	55	36.16±5.53	45.49±9.54	18.31±4.92
	t		-0.258	0.642	-1.217
	P		0.797	0.522	0.226
After treatment	Group-A	59	6.40±1.49^a^	7.68±2.07^a^	3.93±1.22^a^
	Group-B	55	10.25±2.66^a^	10.11±2.94^a^	5.72±1.43^a^
	t		-9.448	-5.063	-7.163
	P		<0.001	<0.001	<0.001

***Note:*** Compared with the same Group-Before treatment,

a P<0.05.

Similarly, no statistically significant difference in FeNO and 25 (OH) D was found between the two groups before the treatment (*P*>0.05). After the treatment, there was a decrease in FeNO in both groups. Post-treatment FeNO levels were significantly lower in the Group- A compared to Group-B (*P*<0.05). Post-treatment 25 (OH) D levels increased compared to before the treatment (*P*<0.05), and were significantly higher in the Group-A (*P*<0.05) (Table-V).

**Table-V:** Comparison of Two Groups of FeNO and 25 (OH) D.

Time	Group	n	FeNO (ppb)	25 (OH)D
Before treatment	Group-A	59	33.68±6.36	13.38±3.41
	Group-B	55	34.89±7.36	14.07±3.58
	t		-0.943	-1.053
	P		0.348	0.295
After treatment	Group-A	59	15.40±5.34^a^	19.59±3.54^a^
	Group-B	55	21.00±5.98^a^	16.22±3.68^a^
	t		-5.277	4.983
	P		<0.001	<0.001

***Note:*** Compared with the same Group-Before treatment,

a P<0.05.

## DISCUSSION

The results of this study indicate that the use of Vitamin-D in the treatment of CODP, in addition to the inhaled BF and TSG sustained-release tablets, has a high clinical value. It can improve lung function, effectively regulate blood gas status, and inhibit the development of inflammation. Formoterol is a receptor agonist drug that can comprehensively regulate the relaxation function of bronchial smooth muscle.^13^ Budesonide has similar effects to glucocorticoids and can effectively alleviate inflammatory response and prevent worsening of the condition.^14^ TSG sustained-release tablets promote the relaxation of bronchial smooth muscle, reduce the degree of bronchospasm, and alleviate symptoms related to airway airflow restriction.^8^,^9^ The combination of these three agents, therefore, plays a synergistic role in improving the patient’s pulmonary ventilation function.^15^

In recent years, clinical studies have found a close correlation between Vitamin-D and COPD.^16^ Research suggests that Vitamin-D can affect COPD through multiple pathways. Vitamin-D may exert a stimulating effect on epithelial cells, increase the content of related antimicrobial peptides such as defensins, strengthen their ability to clear pathogens in the body, and reduce airway hyperresponsiveness.^10^ Moreover, insufficient Vitamin-D content may affect the Th1/Th2 balance, leading to immune dysfunction, that in turn may affect lung function, and exacerbate COPD.^10^,^17^ Additionally, large aggregation of inflammatory cells can potentially trigger a cascade reaction, upregulating inflammatory factors such as IL-8 and IL-12, which are involved in the pathogenesis and progression of COPD. Vitamin-D can reduce the level of NF-kB, strengthen the phagocytic ability of phagocytes, reduce the production of inflammatory factors and proteases, and alleviate the degree of lung tissue damage.^17^,^18^ Yang H et al.^19^ confirmed that intervention with Vitamin-D in patients with COPD on the basis of routine treatment can effectively improve lung function, upregulate immune function related indicators, and help alleviate the severity of the patient’s condition. Li X et al.^20^ conducted a randomized controlled trial on the application value of Vitamin-D in COPD, and showed that Vitamin-D can enhance FEV_1_, FEV_1_/FVC in patients with COPD, prolong six Minute Walk Test, reduce sputum volume, alleviate the overall severity of the condition, and prevent acute exacerbations. This is consistent with the results of this study. Rafiq R et al.^16^ also pointed out that Vitamin-D acts as immune regulator and has anti-inflammatory and antibacterial effect. It can prevent the worsening of COPD and may ensure better disease outcomes. Additionally, Vitamin-D can improve the hormone tolerance of COPD, alleviate the inflammatory response of alveolar macrophages, and regulate the phagocytic function of alveolar macrophages.^16^,^20^ However, Chen FY et al.^21^ showed that both long-term and short-term supplementation of exogenous Vitamin-D did not have a positive effect on the decline of lung function in patients with COPD. This is somewhat different from the results of this study, and may be related to the variability in overall condition of the selected patients and the duration of intervention.

Liu X et al.^22^ pointed out that the onset and progression of COPD are closely related to the inflammatory response. The results of our study indicate that the combination of Vitamin-D, BF, and TSG sustained-release tablets can reduce the expression of inflammatory factors in patients with COPD, which is beneficial for alleviating the degree of inflammatory response. We may speculate that this effect is due to the specific binding of Vitamin-D to Vitamin-D receptors that indirectly or directly acts on lymphocytes (especially T lymphocytes), inhibiting their proliferation and differentiation function and reducing the degree of inflammatory response. 16,19,22,23

In addition, the current study found that the combination of Vitamin-D, BF, and TSG sustained-release tablets can effectively regulate the blood gas status, FeNO, and 25 (OH) D of patients with COPD, which we hypothesize to be a synthetic effect all these three drugs. Feng et al reported that the FeNO level may accurately predict the efficacy of inhaled corticosteroid in the treatment of patients with COPD overlap syndrome.^24^ Uluçoban et al also reported that patients with COPD are in a vicious circle of worse lung function due to vitamin D deficiency, and vitamin D supplementation may improve their lung functions.^25^ Theophylline has also been shown to improve FEV1, lower PaCO_2_, and increase PaO_2_.^26^

### Limitations:

This is a single-center retrospective analysis, with a sample size of only 114 cases and a selection bias. Additionally, our data is limited to the information input into the electronic medical record system and has certain deviations. No prognostic analysis has been conducted, and the long-term value of Vitamin-D combined with BF and TSG sustained-release tablets in the treatment of COPD needs to be verified.

## CONCLUSION

The combination of Vitamin-D, BF, and TSG sustained-release tablets can more effectively improve lung function, regulate patient blood gas status, regulate FeNO, 25 (OH) D, downregulate inflammatory factor levels, and reduce the severity of inflammatory response in the treatment of COPD.

### Authors’ contributions:

**XN:** Conceived and designed the study.

**SZ**, **CW**, **SC**, **JH** and **SZ:** Collected the data and performed the analysis.

**XN:** Was involved in the writing of the manuscript and is responsible for the integrity of the study.

All authors have read and approved the final manuscript.
